# Dietitians’ practices and perspectives of the delivery of nutritional care to cancer survivors in the primary care setting

**DOI:** 10.1007/s00520-025-09330-y

**Published:** 2025-03-17

**Authors:** Henriette G. Ryding, Roshan R. Rigby, Elizabeth A. Johnston, Rozanne Kruger, Lana J. Mitchell

**Affiliations:** 1 School of Health Sciences and Social Work, Griffith University, Gold Coast, QLD Australia; 2 Faculty of Health Sciences and Medicine, Bond University, Gold Coast, QLD Australia; 3 Viertel Cancer Research Centre, Cancer Council Queensland, Fortitude Valley, QLD Australia; 4 School of Exercise and Nutrition Sciences, Queensland University of Technology, Kelvin Grove, QLD Australia; 5 Population Health Program, QIMR Berghofer Medical Research Institute, Herston, QLD Australia; 6 School of Sport, Exercise and Nutrition, Massey University, Auckland, New Zealand

**Keywords:** Dietary management, Dietetics, Survivorship, Supportive care, Referral pathways

## Abstract

**Purpose:**

The number of people living longer after a cancer diagnosis is increasing. Guidelines for cancer survivorship recommend a healthy diet and maintaining a healthy weight post-treatment. While cancer survivors often express the need for professional support for nutrition management, few report seeing a dietitian. This study aimed to explore primary care dietitians’ experiences, practices, and perspectives in providing nutritional care to cancer survivors in Australia.

**Methods:**

This qualitative study used in-depth, semi-structured interviews with primary care dietitians working in private practice and community care. Interviews were recorded and transcribed. A qualitative descriptive methodological approach integrated with a working analytical framework was utilized for coding and data analysis.

**Results:**

Twenty-four dietitians working in primary care participated. Four themes and 13 sub-themes were identified: (1) diversity in dietetic practice and cancer-related care interactions; (2) accessing referral pathways and funding sources in a complex healthcare system; (3) the application of nutrition education, and upskilling in cancer care; (4) client barriers and dietitians' challenges and factors influencing confidence in cancer care.

**Conclusion:**

Dietitians in this study highlighted the need for clear referral pathways to primary care particularly as a continuation of cancer-related care following the acute setting. There is a need for tailored support for dietitians supporting people diagnosed with cancer in the primary care setting, including opportunities to upskill in cancer care.

**Supplementary Information:**

The online version contains supplementary material available at 10.1007/s00520-025-09330-y.

## Background

As cancer detection and treatments improve, more people are living longer after a cancer diagnosis [1]. Many cancer survivors experience long-term physical effects from cancer or its treatment, such as fatigue, poor appetite and taste changes, which can impact diet and nutritional status through weight changes, reduced food intake, and anemia [2, 3]. Cancer and its treatment can also increase the risk of developing additional health conditions, such as cardiovascular disease and diabetes and many cancer survivors may also have a chronic disease prior to their diagnosis [4]. Accordingly, international guidelines for cancer survivors emphasize the importance of healthy lifestyle behaviors post-treatment to prevent chronic disease, reduce cancer recurrence, and improve survival and quality of life [5, 6]. Despite these guidelines, cancer survivors commonly report unmet needs for information on diet, lifestyle, and physical activity; practical skills for healthy eating; dietary strategies for managing ongoing symptoms; and professional support with weight management [7–9]. In Australia, a country with one of the highest survival rates worldwide, the optimal care pathways provide recommendations for best practice care across 18 cancer types [10]. These pathways recommend referral to appropriate multidisciplinary support, such as dietitians, for support with healthy lifestyle behaviors and managing any long-term physical effects. Public and private healthcare systems in Australia recognize dietitians credentialed as Accredited Practising Dietitians (APDs) [11]. In primary care settings, which includes private practice, public health, and community settings, dietitians can support cancer survivors in managing the impact of cancer and its treatment [12] using the Nutrition Care Process, a standardized framework consisting of four steps: assessment, diagnosis, intervention, and monitoring and evaluation [13]. A recent review identified that nutritional interventions from primary care dietitians are effective for intentional weight and fat loss, particularly among breast cancer survivors [14]. Few studies in this review investigated the effectiveness of dietitian support on diet quality. However, growing evidence demonstrates the benefits of improved diet quality for cancer survivors [15]. Further, qualitative and intervention studies have identified that providing follow-up nutritional education after cancer treatment can lead to positive changes in nutritional status for people at risk of malnutrition [16] and symptom improvement in colorectal cancer patients [17]. Cancer survivors also report a need for dietetic support [18] and difficulties accessing allied health care [19].

Despite the need for, and benefit of, dietetic support post-cancer treatment, few cancer survivors report seeing a dietitian [20, 21], with those who did see a dietitian, likely to have been overweight and perceived themselves in poor health prior to their cancer diagnosis [20]. Others considered timing to be an important factor, suggesting that nutrition support is more beneficial when held closer to end of treatment [21]. To date, no studies have investigated the perspectives of primary care dietitians on providing this care, or examined current practices related to referral pathways and funding sources from both public and private healthcare systems. The aim of this study was to explore primary care dietitians’ experiences, practices, and perspectives in providing nutritional care to cancer survivors in Australia. Key objectives were to (1) understand the referral pathways of cancer survivors accessing primary care dietetics services, (2) understand how primary care dietitians provide nutritional management for cancer survivors, and (3) explore the perceived preparedness of primary care dietitians to support cancer survivors.

## Methods

### Study design

Semi-structured interviews were used to explore the experiences, perspectives, and current practices of primary care dietitians who provide care to clients with cancer, defined as people with a current or previous diagnosis of cancer. A qualitative descriptive methodological approach [22] underpinned by relativist ontology and interpretivist-constructivist epistemology [23] guided the research. Ethical approval was received from Griffith University Human Research Ethics Committee (reference: 2024/121). This study is reported according to the consolidated criteria for reporting qualitative research (COREQ) [24]. The first author (H.R.) is an Accredited Practising Dietitian (APD) and a PhD candidate with qualification and experience in qualitative research. Co-authors are PhD-qualified researchers and APDs with extensive experience conducting qualitative research to understand and improve health service and delivery.

### Study setting and recruitment

Australia-based dietitians working in primary care, defined as non-hospital settings such as private practice, public health, and community care, were invited to participate. Participants were not required to have specific experience in cancer nutrition care; however, all had previously consulted with clients who had a personal history of cancer. To maximize diversity, participants were recruited via convenience and snowball sampling through a monthly e-newsletter by Dietitian Connection (an online community for dietitians and nutrition professionals), Cancer Council Queensland’s social media and Health Professionals Cancer Network e-newsletter, and the researchers’ networks and social media. Some participants were recruited directly via websites to target locations that had been underrepresented. In order to mitigate researcher bias owing to participants knowing the researchers prior to the study, reflexivity through critical reflection would be employed to assess how background and prior relationships might influence interactions [25]. Strategies such as maintaining a neutral, open-ended questioning style, seeking diverse perspectives, and critically reviewing interview transcripts for potential biases would be utilized. Interested individuals were invited to view the participant information statement via an online link, and to complete a short survey to capture their demographic and service characteristics. Potential participants were then contacted via email to arrange a 30-min interview at their convenience. Participants were provided with the interview questions in advance, allowing the opportunity for considered responses. Verbal consent was obtained before each interview. Participants received a $50 gift card as an acknowledgement of their time.

### Data collection

During recruitment, participants completed a nine-question online survey covering years of dietetic experience, average duration of initial and review consultations, and postcodes. Geographic work location(s) were then applied using the Modified Monash Model 2019 [26]. In-depth, semi-structured interviews, guided by a protocol developed by the research team, included four overarching topics relating to participants’ referral sources, knowledge, skills, and confidence in cancer care (Table 1). Probing questions were included to gather deeper insights and richer responses. These prompts were refined following a pilot interview with an experienced advanced APD. Interviews were conducted by the first author from March to July 2024 via Microsoft Teams and/or telephone. Member checking was used by allowing participants to review and modify their transcripts [27]. Four participants made minor adjustments for clarification. Assumption of confirmation of transcript accuracy was accepted if no response was received.

**Table 1:** Inquiry logic validating interview questions in relation to study questions

Inquiry logic over four overarching topics	Interview questions
Participant characteristics	
Explores the current roles of participants, providing context and establishing their experience with cancer survivors and confirms participant eligibility	• How many years’ experience do you have as a dietitian (in any field)? • How many years’ experience do you have in primary care (private practice or community health)? • Postcode of your main work location as a primary care dietitian? • Can you give me a brief overview of your role as a primary care/community dietitian?
Referral pathways and nutritional management	
Quantifies time allocation	• How long do you provide for an initial consultation? • How long do you provide for a review consultation?
Permits understanding of the caseloads pertaining to cancer survivors	• How many clients do you consult per week (on average)? • How many clients do you see per month (on average) who have a current or previous cancer diagnosis? • Do you see you see clients during or after the active treatment phase?
Establishes participant familiarity with cancer survivors	• Can you tell me more about the clientele you see for cancer survivorship? • What is the most common type of cancer you see in your patients? • What do cancer survivors primarily want your support with?
Identifies and quantifies referral and funding sources, including self-referral, GP, NDIS, DVA, CDM, CHSP)	• Can you tell me about the referral sources cancer survivors generally use to get to you? • What is your primary referral source for clients with a cancer diagnosis? • What proportion/ how many clients come through this pathway each month?
Explores GP CDM plan in reference to cancer survivors and communication channels	• On average, how many sessions do GPs allocate cancer survivors to see a dietitian through the chronic disease management plan? • Is this number of visits sufficient to reach client goals? • When you are referred a cancer survivorship client, what are they generally referred for? • How do you find communication channels between you and the referring GP/specialist?
Provides understanding of how cancer survivors enter, experience, and exit dietetic services	• I would like you to think of a typical cancer survivor you have seen. Can you describe their journey through your practice?
Perceived knowledge and skills	
Explores participant preparedness in the management of cancer survivors	• How confident are you in the nutritional management of cancer survivors? • What resources do you use when providing nutritional management/advice to cancer survivors? • Do you use guidelines in your practice?
Investigates barriers that may occur during management of cancer survivors	• Can you talk about any challenges you’ve experienced, if any, in providing nutrition management/advice to cancer survivors? • Has working with cancer survivors challenged you on an emotional level? • Have you completed any activities/training to enhance your skills in the nutritional management of cancer survivors? • In what ways do you feel that care for cancer survivors can be improved?
Conclusion	
Provides participants with the opportunity to provide further information	• Would you like to make any other comments regarding the treatment/management of cancer survivors by primary care dietitians?

*GP* general practitioner, *NDIS* National Disability Insurance Scheme, *DVA* Department of Veteran Affairs, *CDM* chronic disease management plan, *CHSP* Commonwealth Health Support Program.

### Data analysis

Demographic and service data were extracted from the online survey and analyzed using descriptive statistics. Analysis was data-driven, using a predominantly inductive coding method [28]. The first author applied the framework method [29] to each interview transcript through seven iterative stages: (1) transcription, (2) familiarization, (3) coding, (4) developing an analytical framework, (5) applying the framework, (6) charting data into a matrix, and (7) interpreting the data. All interviews were recorded, transcribed verbatim using Microsoft Teams, and checked for accuracy alongside the audio recording (transcription). The first author repeatedly read the interviews to familiarize themselves with the data (familiarization), then started coding the interview transcripts concurrently with data collection. Following 24 interviews and recruitment of a diverse sample of primary care dietitians achieved, concurrent data analysis revealed that saturation had been reached, as the research aims had been adequately explored, with sufficient breadth and depth across the working themes [30]. A duplicate coding round was completed for four transcripts, with the remaining research team members each coding a transcript (coding). All researchers collaboratively agreed on preliminary coding labels, forming a working analytical framework, which the first author used to code all transcripts (developing the framework). NVivo 14 was used to highlight and attach quotes to relevant codes, while field notes taken during interviews helped contextualize the data (applying the framework). Once coding was complete, the first author created a matrix outlining key themes, subthemes, and verbatim quotes, which was reviewed by the research team (charting data). Themes and subthemes were refined and condensed through repeated consultations until consensus was reached. Illustrative quotes for each theme and subtheme were chosen, presented alongside the participant number, practice type (e.g., private practice, community), years of experience, and geographical classification (interpreting).

## Results

Twenty-eight primary care dietitians expressed interest in this study, with 24 completing an interview (range: 18–50 min; average interview time: 34 min). One participant preferred a telephone interview. Two participants who were no longer working in primary care answered interview questions based on previous experience. Five participants were known to the first author through researcher networks prior to study commencement. Table 2 summarizes participant characteristics. Participants were located in six states of Australia, with just over half from Queensland (*n* = 13). The majority were female (*n* = 22), worked in private practice (*n* = 22), and in metropolitan areas (*n* = 18). Around half had 3 to < 10 years of experience working as a dietitian in general (*n* = 12) and specifically in primary care (*n* = 13). Most dietitians provided 45 to < 60-min initial consultations (*n* = 17) and 30 to < 45-min review consultations (*n* = 13). Most dietitians consulted with 1–6 clients per month who had a current or previous cancer diagnosis (*n* = 15).

**Table 2:** Characteristics of interview participants (*n* = 24)

Characteristics	*n*	%
Location		
Queensland	13	54
New South Wales	4	17
Victoria	3	13
South Australia	2	8
Western Australia	1	4
Tasmania	1	4
Geographical classification ^a^		
Metropolitan area (metro)	16	67
Regional centre (regional)	5	21
Remote community (remote)	2	8
Small rural town (rural)	1	4
Gender		
Female	22	92
Male	2	8
Years working as a dietitian		
< 1 year	1	4
1– < 3 years	3	13
3– < 5 years	7	29
5– < 10 years	5	21
10– < 15 years	4	17
15– < 20 years	2	8
20 + years	2	8
Years working as a primary care dietitian		
< 1 year	2	8
1– < 3 years	5	21
3– < 5 years	6	25
5– < 10 years	7	29
10– < 15 years	1	4
15– < 20 years	2	8
20 + years	1	4
Length of time provided for an initial consultation		
30– < 45 min	2	8
45– < 60 min	17	71
> 60 min	5	21
Length of time provided for a review consultation		
< 20 min	1	4
20– < 30 min	6	25
30– < 45 min	13	54
45– < 60 min	4	17
> 60 min	0	0
Number of clients consulted per week		
< 10	4	17
11–20	7	29
21–30	4	17
31–40	2	8
41–50	4	17
51–60	2	8
61–100	1	4
Number of clients per month with a current or previous cancer diagnosis		
1–2	5	21
3–4	7	29
5–6	3	13
7–8	2	8
9–10	2	8
> 10	5	21

^a^Postcodes and the Modified Monash Model 2019 were used to determine geographic classification

**Fig. 1:**
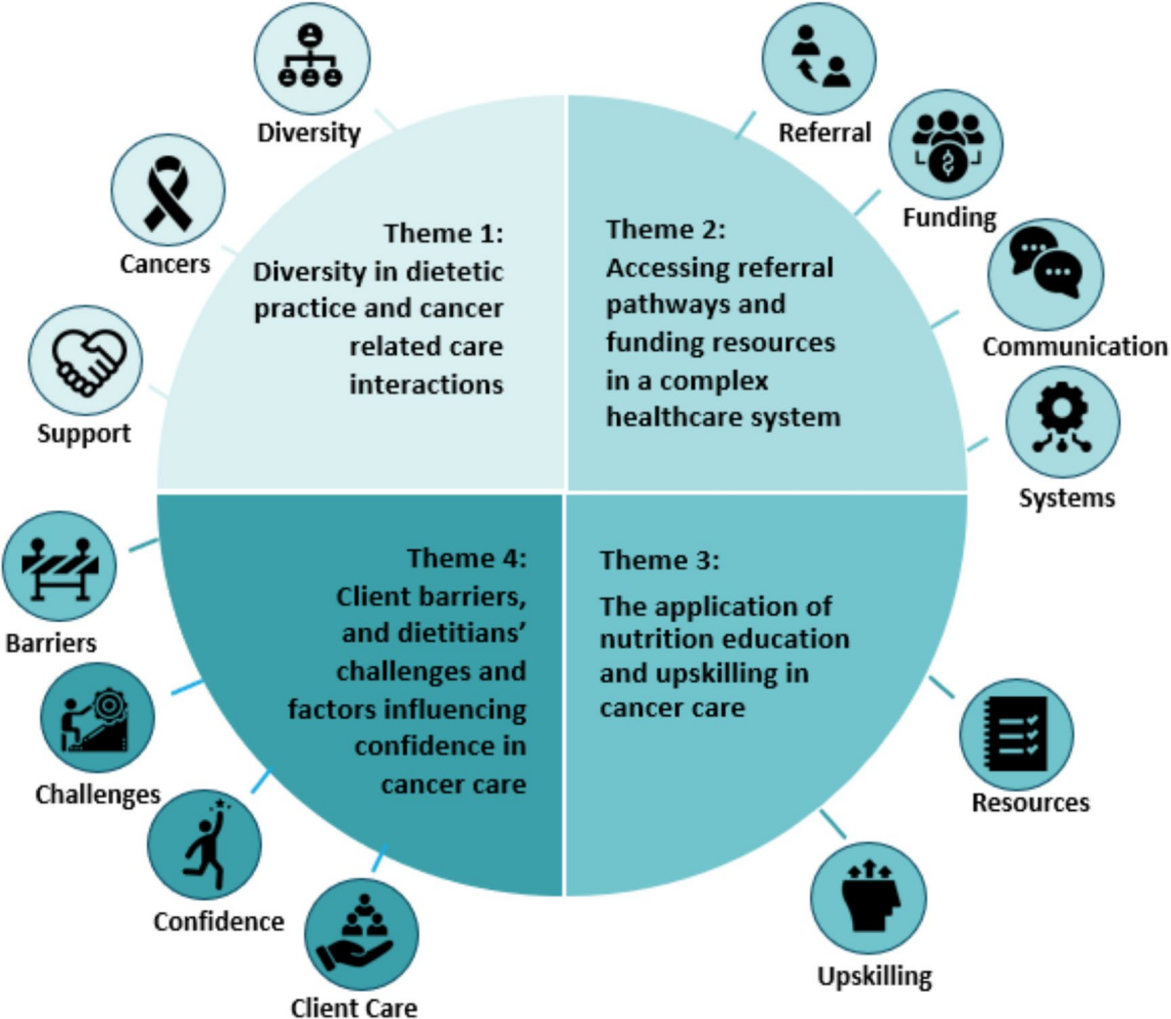
Themes and sub-themes identified through inductive thematic analysis

Four themes and 13 sub-themes were identified: (1) diversity in dietetic practice and cancer-related care interactions; (2) accessing referral pathways and funding sources in a complex healthcare system; (3) the application of nutrition education and upskilling in cancer care; and (4) client barriers and dietitians’ challenges and factors influencing confidence in cancer care (Fig. 1).

### Theme 1: Diversity in dietetic practice and cancer-related care interactions

Theme 1 highlighted the diverse nature of cancer care within primary care dietetic practice. The scope of care varied significantly from one practice to another, influenced by factors such as location, the dietitian’s experience, and the level of specialization.

#### Dietitians show diversity in specializations and practice types

Most dietitians in this study identified as generalists. Others specialized in chronic disease management, bariatric care, sports nutrition, eating disorders, and women’s health. Many managed diverse caseloads across multiple practice locations.

*‘I see patients five days a week in a General Practitioner’s (GP) clinic, and I receive referrals from the community, from GPs and from specialists, and then I also consult at an aged care facility for half a day a week.’ (P10, PP, 3-*<*5 years, 5–6 clients w/ cancer per mth, Remote)*

Some participants brought extensive cancer care experience from previously working in oncology settings: *‘I originally came from the public hospital, and I have worked as a senior oncology dietitian for quite a while.’ (P22, PP, 5-* < *10 years,* > *10 clients w/ cancer per mth, Metro).* Others with generalist backgrounds or different interests reported having smaller cancer-related caseloads.

***‘****A lot of the people I would see with cancer, it just ... comes up in their medical history. So, they might be referred for low nutrition, weight loss, dysphasia, those types of things, not necessarily cancer-specific, but obviously that affects one or the other.’ (P2, PP, 3-*<*5 years, 3–4 clients w/ cancer per mth. Metro)*

#### Dietitians tend to treat clients for a range of cancer types throughout all stages of the cancer journey

Dietitians encountered a range of cancers, with breast and prostate cancer being the most common. Most saw clients after active treatment, though some worked with clients during treatment or with those who developed cancer while under their care: *‘So, I would say probably 70% come to me throughout … during treatment and continue on into survivorship and 30% would come purely just post cancer treatment.’ (P17, PP, 10–15 years,* > *10 clients w/ cancer per mth, Metro).* Some dietitians treated clients with a history of cancer who initially sought help for non-cancer-related reasons. This applied to generalist dietitians with less cancer-specific experience, who were also less likely to attract clients seeking cancer-related support.

#### Clients affected by cancer seek dietetic support for a variety of reasons

Dietitians identified weight-related concerns as most common, especially for clients who had gained weight due to cancer-related therapies. Dietitians noted that post-treatment weight loss could be challenging, especially for those on long-term medication. Unintentional weight loss during cancer treatment, due to factors such as nausea and taste changes, was also common. Clients also sought dietetic care for a variety of reasons, including reducing the risk of cancer recurrence, managing energy levels/ fatigue, accessing oral nutrition supplements, as well as: ‘*optimizing nutritional well-being for cancer outcomes, vitality, longevity, quality of life, [and] symptom control.’ (P17, PP, 10–15 years,* > *10 clients w/ cancer per mth, Metro).*

### Theme 2: Accessing referral pathways and funding sources in a complex healthcare system

Theme 2 examined access to dietetic care for clients with cancer, highlighting the various pathways influenced by healthcare settings, individual needs, and funding models.

#### Clients seek dietitians through numerous referral pathways

Dietitians reported receiving referrals primarily from GPs: *‘I generally get my referrals from GPs. I've had a few, privately self-referred, but yeah, mainly through the GPs through their chronic disease plan.’ (P11, PP, 1-* < *3 years, 9–10 clients w/ cancer per mth, Remote).* Although some were directly referred by specialists or oncologists: *‘Predominantly in terms of a referral for an oncology diagnosis, it would be directly from the oncologist or through being involved with local community oncology services.’ (P24, PP, 20* + *years,3–4 clients w/ cancer per mth, Regional).* In multidisciplinary practices, dietitians worked alongside other health professionals, benefiting from diverse referral sources within the same practice: *‘The other one would be in-house referrals. So, if they've started training with an ex-phys [exercise physiologist], or if they're seeing a physio[therapist], then sometimes it's referrals from them.’ (P3, PP, 1-* < *3 years, 1–2 clients w/ cancer per mth, Metro).*

Clients also self-referred to dietitians, identified through word of mouth or online research. Some dietitians reported receiving referrals by collaborating with cancer organizations and through local cancer support groups. Community dietitians, primarily funded through government programs, provided affordable or free-of-charge services, particularly to vulnerable populations. This was mostly for a limited time post-treatment, with clients referred to dietetic care after meeting eligibility criteria and triaging based on need: ‘*We have an “unrestricted” number of visits, as many as the patient requires to achieve their goals.’ (P5, Community, 3-* < *5 years, 3–4 clients w/ cancer per mth, Metro).*

#### Funding sources used by clients both hindered and enabled access to dietetic care

Several funding sources are available for accessing primary care dietetic services, including government programs and private health insurance, each offering different levels of coverage depending on the individual’s circumstances. General practitioners commonly used chronic disease management plans and team care arrangements, and dietitians typically received referrals that covered two to three visits. Many dietitians felt that this was insufficient to fully achieve client goals: *‘Often dietetics ends up being like only two sessions, which definitely isn't enough to make big changes.’ (P3, PP, 1-* < *3 years, 1–2 clients w/ cancer per mth, Metro).*

Some dietitians charged only the amount refundable by Medicare, Australia’s universal health insurance scheme, making dietetic care more accessible for clients facing financial difficulties. However, even with this support, some clients were still unable to complete their full course of care:* ‘With Medicare, it is a fully bulk billed service, so a lot of them don't want to then pay the additional, once they have… reached the threshold for their referrals*.*’ (P11, PP, 1-* < *3 years, 9–10 clients w/ cancer per mth, Remote).*

Clients were responsible for funding any visits beyond the subsidized sessions, either out of pocket or through private health insurance if they had coverage. Some clients initially relied on Medicare subsidies, and once those were exhausted, used private health insurance to cover additional sessions:* ‘Normally it would be one or the other and then, yes, if they've run out of Medicare, would move on to private health.’ (P23, PP, 5-* < *10 years, 1–2 clients w/ cancer per mth, Regional).* Dietetic consultations were also funded through national disability and veteran support programs.

#### Dietitian perceptions of interdisciplinary communications

When receiving referrals from GPs and other healthcare professionals, such as oncologists, clear, consistent, and timely communication, was needed to ensure cohesive client care, particularly when all parties were not within the same practice. Most dietitians found coordinating care challenging, particularly when there was limited time between receiving referrals and scheduled client appointments. Almost all dietitians reported suboptimal outcomes when communicating with GPs and some with oncologists. Much of the communication was in written form, presenting challenges including insufficient client information in referral letters and delays in receiving necessary test results prior to consultations. This hindered collaborative care and impacted dietitians’ ability to provide timely, well-informed consultations. Dietitians used phone calls when urgent communication was needed to ensure client safety and optimal care. However, this method was often inefficient, with dietitians frequently unable to contact the GP.

*‘I feel I do most of the communicating. Letters and the constant follow-ups… I don't often get communication in return. Which is upsetting at times just because it's such a tricky area, and unless I ask, I don't often get communication.’ (P19, PP, 3-*<*5 years, 5–6 clients w/ cancer per mth, Metro)*

#### Systemic improvements within the healthcare system are needed to enhance coordination and quality of care

Many dietitians expressed a strong desire for greater involvement in multidisciplinary teams, indicating that collaborating closely with other healthcare professionals would enhance patient care and support more comprehensive treatment plans.

*‘I think it's also about having a community who can talk to each other, who are linked in with each other and you, I guess, would have a better ability to manage a client as a community as opposed to as singular medical professional doing their job, because there is crossover and there are things that require two or three people to have input in and I guess you probably just don't have that in this space either.’ (P22, PP, 5-*<*10 years,* >*10 clients w/ cancer per mth, Metro)*

Dietitians emphasized the need for improved coordination between clients, funding bodies, and healthcare professionals to ensure seamless and effective care.

*‘I think just having that pathway in place that everyone with a cancer diagnosis is at least offered the opportunity to see a dietitian would help the patient, and it helps dietitians as well because you're then not seeing people five years down the line that are overwhelmed and have been told 20 different things by doctors and the internet and naturopaths and physios and people that are just trying to help that have just added to the confusion.’ (P10, PP, 3-*<*5 years, 5–6 clients w/ cancer per mth, Remote)*

Dietitians called for greater GP education on the role of dietetic care in cancer survivorship, emphasizing that awareness of the importance of nutrition in cancer care and the dietitian’s contribution could enhance referral processes, collaboration, and client outcomes.

***‘****GPs will often not even think to send a patient to a dietitian unless they were really malnourished and by that stage they’re already malnourished. So, it would probably be helpful to have some sort of education for GPs on how dietitians can help their patients because I think in the community, there are a lot of people very interested in nutrition.’ (P8, PP, 3-*<*5, 5–6 clients w/ cancer per mth, Metro)*

### Theme 3: The application of nutrition education, and upskilling in cancer care

Theme 3 examined the use of cancer nutrition education in practice and the need for upskilling in cancer care.

#### Nutrition resources and the use of guidelines in cancer care

Dietitians reported using printed educational materials from various sources to support clients with cancer and to refresh their own knowledge. Most dietitians relied on Nutrition Education Materials Online (NEMO) resources (designed to support dietitians), often combining them with materials from Cancer Council, eviQ (online cancer resource), Peter MacCallum Cancer Centre, or Practice-based Evidence in Nutrition (PEN) resources. When appropriate resources were unavailable, dietitians reported creating their own, particularly when a highly individualized approach was needed.

*‘I’ll often use the ESPEN guidelines for cancer care. I have the [Peter MacCallum Cancer Centre] oncology nutrition book, whether it's like the COSA head and neck guidelines, even the [Peter MacCallum] Can Eat Pathway to prevent malnutrition.’ (P22, PP, 5-*<*10 years,* >*10 clients w/ cancer per mth, Metro)*

Many dietitians relied on guidelines and information from the European Society for Clinical Nutrition and Metabolism (ESPEN), PEN, optimal care pathways, Clinical Oncology Society of Australia (COSA), the Peter MacCallum Cancer Centre, and the World Cancer Research Fund (WCRF), to inform their nutritional recommendations for clients with cancer. Dietitians noted that the lack of resources explicitly tailored to cancer survivors in a post-recovery phase in primary care made managing these clients challenging.

*‘There is a lot of emphasis on nutrition care for patients during active treatment, but not many nutrition guidelines available for cancer survivorship and nutrition management. I believe … there was the ESPEN guidelines, but they focused on very broad recommendations such as consume more whole grain, fruit, vegetables, reducing fat, red meat, alcohol etc.- basically to just follow the healthy Australian guidelines. Maybe this is enough? I don’t know. ‘(P9, Community, 10–15 years,* >*10 clients w/ cancer per mth, Metro)*

Some dietitians noted that guidelines alone were insufficient without experience in oncology care, as they could be difficult to apply to each client’s unique needs. Many dietitians did not use cancer-specific guidelines, mainly due to the small size and nature of their cancer caseload, relying instead on the Australian Dietary Guidelines or the Mediterranean diet recommendations.

#### Upskilling in cancer care is important for dietitians wanting expert knowledge

Dietitians described upskilling in cancer care through participating in professional development activities like webinars, seminars, and educational sessions. Many expressed a desire to upskill in cancer care: *‘I think, no matter where you are at in dietetics…it will almost be inevitable that you come across someone with a history of cancer. So, it is important to upskill in this area.’ (P16, PP, 1-* < *3 years, 3–4 clients w/ cancer per mth, Metro).* However, dietitians found it challenging to identify relevant opportunities and reported difficulties in maintaining up-to-date knowledge of various cancer types and treatments and a lack of experience in cancer care: *‘Medications and things … there is always something new coming that you have to kind of look into or get advice on.’ (P10, PP, 3-* < *5, 5–6 clients w/cancer per mth, Remote).* Dietitians also highlighted the absence of opportunities for primary care dietitians in Australia to obtain specialized accreditation in cancer care and felt this limited their ability by not having structured learning pathways to formally develop and demonstrate expertise and high-quality care which may undermine clients’ confidence in professional credibility.

*‘Having services that are cancer-specific, ultimately because I don't think a lot of that exists, and I think people really want to see someone who … actually has the expertise to know what has happened and what that means for you now … because I don't think there is a lot of that tailored support in any allied health profession, including dietetics.’ (P22, PP, 5-*<*10 years,* >*10 clients w/ cancer per mth, Metro)*

Several dietitians described upskilling in other areas, such as behavioral counselling, that they could apply to support clients with cancer. Others were unsure about the relevance of upskilling in cancer care since these clients were a small part of their caseload.

***‘****I'm not sure if it's an area that I do want to upskill then, if I'm not going to be seeing them all that often. I'd rather just refer on to someone who I know is really confident within that area.’(P3, PP, 1-*<*3 years, 1–2 clients w/ cancer per mth, Metro)*

### Theme 4: Client barriers, and dietitians’ challenges and factors influencing confidence in cancer care

Theme 4 examined dietitians’ perspectives on barriers that clients with cancer face when they access dietetic care, and how these may have impacted dietetic practice. It also explored the challenges that dietitians experienced when caring for clients with cancer, including difficulties faced in providing effective nutritional support to this population, highlighting the complexities and obstacles involved in delivering comprehensive care to clients with cancer.

#### Dietitian perspectives on client barriers to accessing primary care dietetic services

Dietitians identified several barriers preventing cancer survivors from accessing primary care dietetic services. These included cancer survivors’ need for dietetic support being met in acute healthcare settings, and a desire to minimize healthcare appointments post-treatment. Dietitians also noted that cancer survivors often had limited knowledge of the services available to them and a lack of direction for continued nutrition care after leaving the hospital setting.

*‘There's no flow on effect … women, multiple years down the track, that are still wanting to address things from two years ago, come in, you know, with multiple things they want to address and it's like, well, we could have if you knew that this was an option for you, and this would have benefited you earlier. You might have been able to resolve this a lot sooner.’ (P6, PP, 5-*<*10 years, 3–4 clients w/ cancer per mth, Metro****)***

Most dietitians felt that financial pressures prevented clients from fully or consistently accessing their services, resulting in fewer sessions than needed and potentially diminishing the effectiveness of dietary guidance: *‘I still saw the need for dietetic intervention in a lot of the patients that fell out and couldn’t come to see me due to the lack of funding.’(P9, Community, 10–15 years,* > *10 clients w/ cancer per mth, Metro).*

A perceived lack of clear and concise information regarding available services and funding in both private and public systems could lead to confusion among clients, further reducing client engagement in dietetic care.

*‘And it's so hard for patients … the last thing they need to deal with is, “Oh well, if I go through this pathway versus this pathway, do I have to get my jejunal feed tube inserted at this public hospital, where I'm going to get an ongoing supply of nutrition that I need, as opposed to if I get it done at this hospital as a private patient then I need to pay for myself”… it's really challenging.’ (P21, PP, 15-*<*20 years,7–10 clients w/ cancer per mth, Metro)*

Dietitians in regional areas reported that travel distances and limited facilities hindered client access, while metropolitan-based dietitians did not report these concerns.

#### Dietitians face challenges in caring for clients with cancer

Many dietitians expressed concern over the rise of alternative treatments, misinformation, and nutrition myths, often spending time debunking these before providing evidence-based advice:* ‘There's a lot of misinformation out there that can save people a lot of hassle if they can just speak to someone that is able to guide them. ‘(P10, PP, 3-* < *5 years, 5–6 clients w/ cancer per mth, Remote).*

Some dietitians, especially those working independently, struggled with limited support for enteral feeding, and many felt a general lack of support for their profession, emphasizing the need for more robust advocacy to secure the role of dietitians in primary care.

*‘Getting the oncology team a little bit more on board to referring to dietitians in the community … and just advocating for dietetics and the importance of nutrition, which I think we need to do more of that.’ (P19, PP, 3-*<*5 years, 5–6 clients w/ cancer per mth, Metro)*

There were concerns regarding poor coordination between hospital and primary care, affecting the continuity of nutritional support for clients with cancer transitioning into the community.

*‘It's a real confusion as to who will still be responsible for your ongoing care. Are we now finished with the oncologist dietitian? And now I'm taking over the ordering of your supplements, and am I your port of call for that? I think that's the real issue, there's no clear-cut handover from the specialist realm to the private practice realm.’ (P6, PP, 5-*<*10 years, 3–4 clients w/ cancer per mth, Metro)*

Some dietitians with oncology expertise worried that their less-experienced colleagues might provide generic, less tailored advice, potentially impacting the quality of nutrition care.

*‘…what we see often is then that the patient feels uncertain about the advice and the care that they've received, and it taints the perception of dietitians… and it's not at all the fault of us as a profession. It’s lack of experience, which you can’t obtain without experience or mentoring… and it's that time and effort that provides the robust care that people value. And if you don't do that, that's when you get that kind of wishy-washy, untailored, just generalized advice that particularly cancer survivors...they don't respond to, they don’t respect it, and it doesn't feel individualized and therefore doesn't feel particularly useful, or it feels like something they could Google themselves.’ (P17, PP, 5-*<*10 years,* >*10 clients w/ cancer per mth, Metro*)

Dietitians also highlighted the emotional challenges of treating clients with cancer, especially when clients experience recurrence or pass away. This emotional burden could be intensified by personal experiences with cancer, whether through themselves, family, or friends. *‘Saying goodbye to your client and never knowing when the last time you’ll see them is going to be. That’s hard, particularly when you’ve developed a relationship with somebody over a period of time.’ (P12, PP, 10–15 years, 7–8 clients w/ cancer per mth, Regional)*

#### Confidence is related to experience in cancer care

Most dietitians reported average to high confidence in cancer care, particularly those with previous work experience or those receiving/giving mentorship in oncology.

*‘Years of experience. I think without that, I wouldn't feel anywhere near that confident. I also feel perhaps more confident because I've got a team around me. So, I'm not working in isolation.’ (P17, PP, 5-*<*10 years,* >*10 clients w/ cancer per mth, Metro)*

Dietitian confidence was lower when dealing with clients undergoing active treatment, head and neck cancers, complex gastrointestinal symptoms, or enteral feeding, and keeping up with new cancer medications and online diet trends. Many dietitians felt their university training lacked a focus on cancer nutrition in primary care, and limited experience with cancer survivors added to their uncertainty. In such cases, dietitians often consulted oncology specialists, searched online, or referred clients to more specialized dietitians.

*‘If I felt that there was something that I just didn't know about or required help with, I tell the client that, and I'd ring a tertiary referral center where the dietitian was specifically treating cancer clients and maybe ask some questions there.’ (P1, PP, 15-*<*20 years,* >*10 clients w/ cancer per mth, Rural)*

#### Client-centered care

Dietitians expressed the importance of holistic, individualized, and inclusive care by tailoring recommendations to each client’s unique needs and preferences. Some highlighted that flexibility in practice was required to maintain a client-centered approach. Dietitians also found that humor, personalized care, and empathy enabled them to build connections with their clients, enhancing their experience of care.

***‘****People don't care how much you know until they know how much you care, and you've got to, you've got to let your personality come through and connect with them as a person as opposed to someone who has a condition that you're trying to provide nutritional advice with.’ (P1, PP, 15-*<*20 years,* >*10 clients w/ cancer per mth, Rural)*

## Discussion

This study is the first to examine primary care dietitians’ experiences, perspectives, and current practices in providing nutritional care to cancer survivors. Most dietitians were generalist private practitioners, working in metropolitan settings, with some specializing in cancer care. Client access to dietetic services was often through GP referrals. Dietitians highlighted that streamlined and affordable care through all referral avenues was essential for enabling cancer survivors to access dietitian support in either private practice or community care after completing active cancer treatment. Dietitians perceived that financial pressures on clients and insufficient Medicare rebates often resulted in an inadequate number of visits, thereby limiting optimal dietetic care for cancer survivors. Multidisciplinary connections were considered ideal for enhancing quality of care, leading to improved client outcomes. Some dietitians sought to acquire cancer-specific skills through professional development opportunities enabling them to more confidently provide nutritional care to cancer survivors.

Australia has one of the best, albeit complex, healthcare systems in the world [31], that combines universal health care (i.e., Medicare) with private health insurance. In this study, primary care dietitians identified GPs as a key source of referrals. In Australia, GPs serve as gatekeepers for Medicare funding, providing eligible clients with referrals to subsidized dietetic care via a chronic disease management plan. This necessitates a GP to generate a GP management plan and team care arrangements with up to five visits available for allocation to allied health practitioners, including dietitians, psychologists, and physiotherapists [32]. With limited appointments available for allied health, it is important that dietitians are equipped with knowledge and confidence to advocate for their role in supporting cancer survivors. A key focus of this advocacy could be building stronger connections with GPs who are a primary referral source.

Primary care dietitians in this study indicated that current government-subsidized systems for primary healthcare, such as those described above, do not allow for sufficient dietitian visits to achieve nutritional goals, limiting the effectiveness of dietetic care for cancer survivors. Further, while some dietitians offered bulk billing (i.e., no out-of-pocket payment because the consultation cost matches the Medicare rebate), the low rebate amount (AUD$60.35 for consultations lasting at least 20 min [33]) and the need for longer consultations meant that dietitians often charged fees beyond the rebate, requiring an out-of-pocket payment by the client. Dietitians in our study perceived that out-of-pocket costs can be a significant barrier for cancer survivors to access dietetic care. This aligns with previous studies which have identified that many people diagnosed with cancer experience financial burden due to the costs of cancer treatment and loss of income, often leading them to forgo or discontinue necessary treatment [34]. There are existing calls to rethink funding models for Australia’s public health system as current models can contribute to uncoordinated, fragmented care, particularly for clients with chronic conditions or ongoing needs [35]. Based on our findings, increasing the number of visits allocated to allied health services could lead to greater engagement with primary care dietitians and improved nutritional outcomes for cancer survivors.

In this study, primary care dietitians perceived gaps in care for cancer survivors following hospital discharge, and many highlighted a lack of referrals and inadequate coordination for post-treatment nutrition care. This is supported by the literature showing that many survivors are not educated about, nor do they receive dietary information and support post-treatment [9], despite clear guidelines emphasizing the role of nutrition in reducing cancer recurrence risk and chronic disease [5]. Furthermore, when cancer survivors have unmet information needs in healthcare settings, they often turn to online sources for nutrition advice [9]. However, previous research has documented that much of the nutrition information available online is not reliable [36]. Our findings show that dietitians in this study reported spending a significant amount of their consultation time debunking myths surrounding cancer nutrition. These findings suggest that better coordination of care between acute settings, GPs, and primary care dietitians is needed for cancer survivors following active cancer treatment.

Many primary care dietitians in this study emphasized the importance of multidisciplinary collaboration and expressed a strong desire to be more involved in such teams for support, leading to better care and improved client outcomes. In particular, dietitians working in private practice or as sole practitioners often felt isolated. This aligns with literature highlighting that high-quality, person-centered care requires personalized, coordinated efforts that address all client needs, including social and mental health while respecting client preferences [37]. This also aligns with guidelines that highlight the benefits of integrating dietetic care with exercise to improve outcomes for cancer survivors [5, 38]. As long-term survivors often have complex medical needs, including multiple co-morbidities [39], multidisciplinary teams can help by sharing technical knowledge and supporting diverse expertise, resulting in improved patient outcomes [40, 41]. More action is needed to improve multidisciplinary collaboration, with a focus on integrating sole practitioners into more collaborative, team-based environments.

Some dietitians reported limited opportunities to upskill and gain accreditation in cancer care. In the USA, Registered Dietitians can earn a Board-Certified Specialist in Oncology nutrition (CSO) credential [42], but this certification is currently not offered in countries like Australia, the UK, and Canada. Consequently, dietitians in Australia, especially those working in primary care, are not able to gain formal recognition for specializing in cancer nutrition. Our study identified that cancer-related professional development opportunities were not always available, and many dietitians noted a lack of resources, especially for primary care-related issues, such as side effects of new or long-term cancer therapies. This perspective aligns with a study of oncology dietitians in Australia that reported needing more professional education and resources for dietary patterns and their relevance in the treatment of cancer-related malnutrition [43]. While there is a need to advocate for improved referrals to primary care dietitians, there is a concurrent need to address the lack of appropriate and accessible educational resources for primary care dietitians to effectively support cancer survivors. Considering that Australia has one of the highest rates of cancer survivorship globally [44], and the number of clients with a personal history of cancer will increase in coming years, there is a need to develop resources for upskilling primary care dietitians in nutrition care for cancer survivors.

### Strengths and limitations

All interviews were conducted by the first author using a semi-structured guide, ensuring consistency in style and process. While the researcher’s background as a dietitian may have introduced unconscious bias, referring to the unintentional influence of their professional experience on data collection and interpretation, it also provided valuable insight into the study’s context. This background provided a deep understanding of dietetic practice, including its standards, challenges, and constraints. Such familiarity allowed for more nuanced questioning, effective probing, and an appreciation of the practical implications of participants’ responses. The use of qualitative research through semi-structured interviews provided rich, in-depth data on primary care dietitians’ experiences. Researcher bias from prior acquaintance with participants was mitigated through approaches such as reflexivity through critical reflection [25]. As a result, this was not considered a significant limitation. Member checking was additionally used to ensure rigor and trustworthiness, whereby participants reviewed their interview transcript to confirm the interview accurately reflected their experiences. Recruitment resulted in a diverse sample of primary care dietitians from across Australia, though most participants were from Queensland, and the two territories of Australia were not represented. Therefore, these findings may not be generalizable to primary care dietitians nationally.

## Conclusion

This study offers novel insights into the experiences, perspectives, and current practices of primary care dietitians in providing nutritional care to clients with cancer. Dietitians highlighted the need for clear referral pathways and improved coordination of care between acute care settings, GPs, and primary care dietitians. Primary care dietitians interested in cancer care sought opportunities to develop expertise and desired more professional education and tailored support to better serve their clients with cancer. As cancer incidence and survival increases, future work needs to focus on enhancing referral pathways with integration across acute and primary care settings, improving professional support and education for primary care dietitians, and developing resources to help primary care dietitians advocate for their role in cancer nutrition care.

## Supplementary Information

Below is the link to the electronic supplementary material.

Supplementary file1 (DOCX 30 KB)

## Data Availability

No datasets were generated or analysed during the current study.
