# Supplementary material for: Dietitians’ practices and perspectives of the delivery of nutritional care to cancer survivors in the primary care setting
Source: Support Care Cancer. 2025 Mar 17;33(4):290. doi: 10.1007/s00520-025-09330-y (PMC11913905; doi:10.1007/s00520-025-09330-y)
Supplement: Supplementary file 1 — Supplementary file1 (DOCX 30 KB) [file 520_2025_9330_MOESM1_ESM.docx]

**Title**: Dietitians’ practices and perspectives of the delivery of nutritional care to cancer survivors in the primary care setting

**Authors:** Henriette G. Ryding, Roshan R. Rigby, Elizabeth A. Johnston, Rozanne Kruger, Lana J. Mitchell

**Affiliations and ORCID ID:**

Ms. Henriette G. Ryding, School of Health Sciences and Social Work, Griffith University, Gold Coast, Queensland, Australia. ORCID ID: https://orcid.org/0000-0002-7502-5259

Dr Roshan R. Rigby, Faculty of Health Sciences and Medicine, Bond University, Gold Coast, Queensland, Australia. ORCID ID: https://orcid.org/0000-0003-4829-8371

Dr Elizabeth Johnston, Viertel Cancer Research Centre, Cancer Council Queensland, Fortitude Valley, Queensland, Australia; School of Exercise and Nutrition Sciences, Queensland University of Technology, Kelvin Grove, Queensland, Australia; Population Health Program, QIMR Berghofer Medical Research Institute, Herston, Queensland, Australia.
ORCID ID: https://orcid.org/0000-0002-9486-5704

Prof Rozanne Kruger, School of Health Sciences and Social Work, Griffith University, Gold Coast, Queensland, Australia; School of Sport, Exercise and Nutrition, Massey University, Auckland, New Zealand. ORCID ID: 0000-0003-2117-3237

Dr Lana J. Mitchell, School of Health Sciences and Social Work, Griffith University, Gold Coast, Queensland, Australia. ORCID ID: http://orcid.org/0000-0002-7892-2131

**Corresponding author:** Lana J. Mitchell [Lana.mitchell@griffith.edu.au](mailto:Lana.mitchell@griffith.edu.au)

**Appendix 1.**

**Consolidated criteria for reporting qualitative studies (COREQ): 32-item checklist**

| **No.** | **Item** | **Description** | **Section #** |
| --- | --- | --- | --- |
| **Domain 1: Research team and reflexivity** | | |  |
| Personal characteristics | | |  |
| *1.* | Interviewer/facilitator | Which author/s conducted the interview or focus group? | Data collection |
| *2.* | Credentials | What were the researcher's credentials? *E.g. PhD, MD* | Study design |
| *3.* | Occupation | What was their occupation at the time of the study? | Study design |
| *4.* | Gender | Was the researcher male or female? | N/A |
| *5.* | Experience and training | What experience or training did the researcher have? | Study design |
| Relationship with participants | | |  |
| *6.* | Relationship established | Was a relationship established prior to study commencement? | N/A |
| *7.* | Participant knowledge of the interviewer | What did the participants know about the researcher? *E.g. Personal goals, reasons for doing the research* | Study setting and recruitment: Participant Information Statement |
| *8.* | Interviewer characteristics | What characteristics were reported about the interviewer/facilitator? *E.g. Bias, assumptions, reasons and interests in the research topic* | Strengths and limitations |
| **Domain 2: Study design** | | |  |
| Theoretical framework | | |  |
| *9.* | Methodological orientation and theory | What methodological orientation was stated to underpin the study? *E.g. grounded theory, discourse analysis, ethnography, phenomenology, content analysis* | Study design |
| Participant selection | | |  |
| *10.* | Sampling | How were participants selected? *E.g. purposive, convenience, consecutive, snowball* | Study setting and recruitment |
| *11.* | Method of approach | How were participants approached? *E.g. face-to-face, telephone, mail, email* | Study setting and recruitment |
| *12.* | Sample size | How many participants were in the study? | Results |
| *13.* | Non-participation | How many people refused to participate or dropped out? What were the reasons for this? | Results |
| Setting | | |  |
| *14.* | Setting of data collection | Where was the data collected? *E.g. home, clinic, workplace* | Data collection |
| *15.* | Presence of nonparticipants | Was anyone else present besides the participants and researchers? | Data collection |
| *16.* | Description of sample | What are the important characteristics of the sample? *E.g. demographic data, date* | Results and Table 1. |
| Data collection | | | |
| *17.* | Interview guide | Were questions, prompts, guides provided by the authors? Was it pilot tested? | Data collection and Table 2. |
| *18.* | Repeat interviews | Were repeat interviews carried out? If yes, how many? | N/A |
| *19.* | Audio/visual recording | Did the research use audio or visual recording to collect the data? | Data analysis |
| *20.* | Field notes | Were field notes made during and/or after the interview or focus group? | Data analysis |
| *21.* | Duration | What was the duration of the interviews or focus group? | Results |
| *22.* | Data saturation | Was data saturation discussed? | Data analysis |
| *23.* | Transcripts returned | Were transcripts returned to participants for comment and/or correction? | Data collection |
| **Domain 3: analysis and findings** | | | |
| Data analysis | | | |
| *24.* | Number of data coders | How many data coders coded the data? | Data analysis |
| *25.* | Description of the coding tree | Did authors provide a description of the coding tree? | Data analysis |
| *26.* | Derivation of themes | Were themes identified in advance or derived from the data? | Data analysis and Results |
| *27.* | Software | What software, if applicable, was used to manage the data? | Data collection and Data analysis |
| *28.* | Participant checking | Did participants provide feedback on the findings? | Data collection |
| Reporting | | | |
| *29.* | Quotations presented | Were participant quotations presented to illustrate the themes / findings? Was each quotation identified? *E.g. Participant number* | Yes. Table 3. |
| *30.* | Data and findings consistent | Was there consistency between the data presented and the findings? | Yes. |
| *31.* | Clarity of major themes | Were major themes clearly presented in the findings? | Yes, all themes were discussed |
| *32.* | Clarity of minor themes | Is there a description of diverse cases or discussion of minor themes? | Sub-themes were discussed |
